# Saliva secretion disorder in a schizophrenic patient - a problem in dental and psychiatric treatment: a case report

**DOI:** 10.1186/s12991-015-0052-4

**Published:** 2015-03-10

**Authors:** Danuta Maria Moś

**Affiliations:** Health Care Centre Euro-Med Bytom, Górnicza 12 Str, Bytom, 41-935 Poland

**Keywords:** Dental plaque, Neuroleptic, Saliva secretion, Schizophrenia

## Abstract

**Objective:**

Saliva secretion disorder may appear in patients at any age and represents a serious problem in interdisciplinary treatment. It is manifested by hyposecretion or hypersecretion of saliva. One of the major groups of patients who have been diagnosed with saliva secretion disorder includes those treated with neuroleptics. Among patients taking neuroleptics, schizophrenic patients represent the least cooperative group in terms of doctor-patient relationship. Schizophrenia is a mental disorder exacerbated by uncontrolled neuroleptic dose reduction or regardless of applied pharmacotherapy.

**Method:**

This paper presents a clinical case of a 30-year-old schizophrenic patient with saliva secretion problems.

**Results:**

In schizophrenia, thought disorders (TD) and social functioning impairment have a negative impact on patients’ somatic health care. Saliva hyposecretion and its health consequences, such as parodontitis and caries, are the reasons why the patients decide to have a dental appointment.

**Conclusion:**

This paper contains important information for dentists, psychiatrists, and psychologists, as it raises an issue of a proper interdisciplinary care approach provided to schizophrenic patients. It emphasises the importance of psychoeducation and draws attention to social functioning of mentally ill patients.

## Introduction

**Figure 1:**
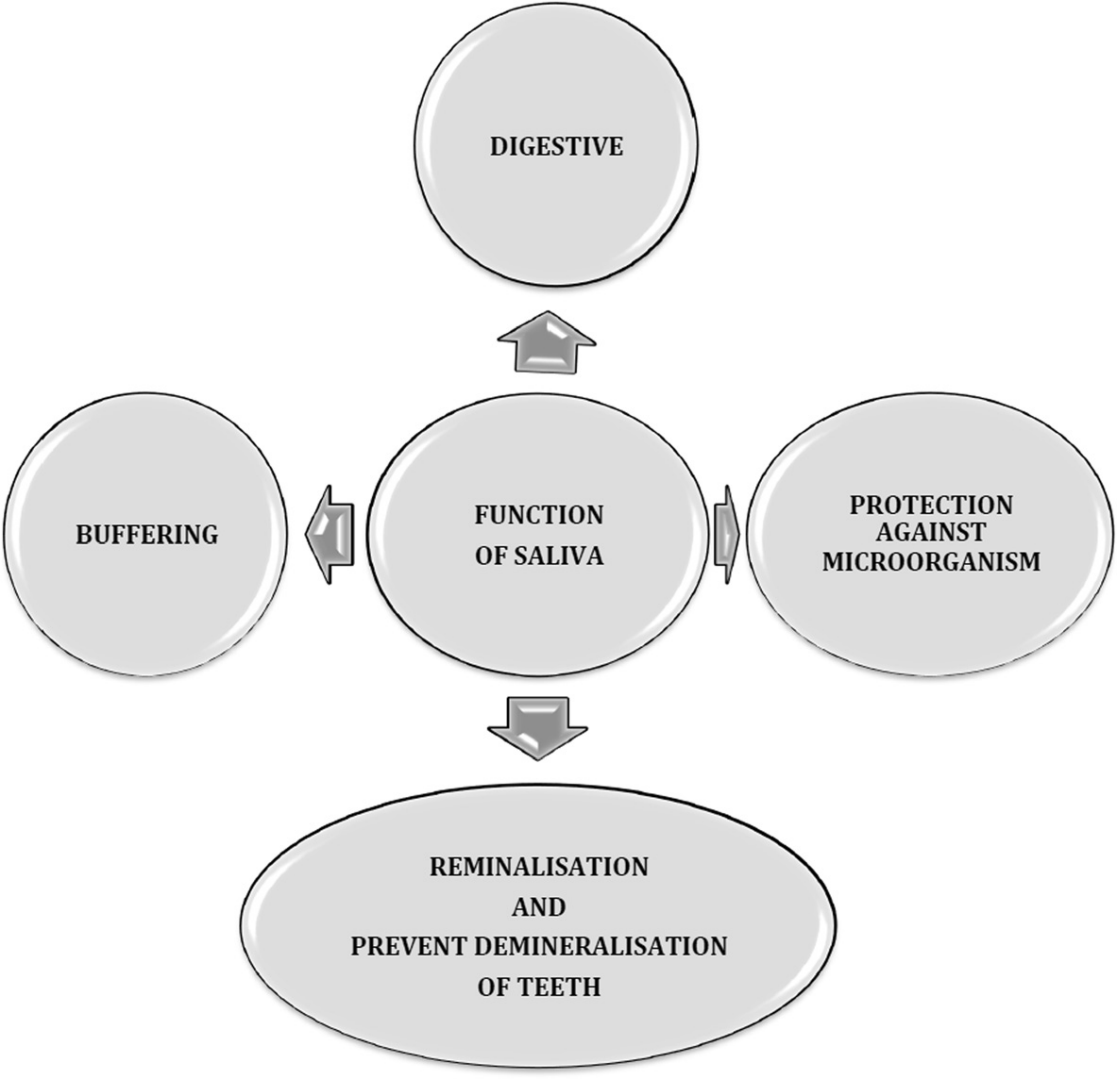
**Function of saliva.**

Salivary fluid is an exocrine secretion consisting of approximately 99% water and a variety of electrolytes such as sodium, potassium, calcium, chloride, magnesium, bicarbonate, and phosphate. Also, this fluid contains proteins, represented by enzymes, immunoglobulins and other antimicrobial factors, mucosal glycoproteins, albumins and some polypeptides, and oligopeptides important for oral health. The components interact and are responsible for the various functions attributed to saliva (Figure 1). Saliva secretion disorder is considered to be troublesome and affects negatively the patients’ health status. It is most frequently related to the use of anticholinergics drugs [1,2]. Schizophrenia is one of the most common disorders in which saliva secretion is disturbed as a result of antipsychotic pharmacotherapy [3]. Schizophrenia is a mental disorder which can be characterised by both positive symptoms (delusions defined as false beliefs and hallucinations defined as false perceptions) and negative symptoms (flat affect, apathy, anhedonia) [4]. Impaired social functioning of schizophrenic patients, manifested by difficulties in recognising their needs and lower ability to communicate in society, constitutes the main behavioral feature of schizophrenia [5,6].

Schizophrenic patients do not always care for their health and personal hygiene, including oral cavity hygiene [7,8]. Their dental health status is poor as compared with mentally healthy people. It is also the consequence of irregular and unhealthy diet and more frequent smoking habits [9]. According to the study, only 42% of schizophrenic patients brush their teeth regularly (at least twice a day) [10]. This poor dental health leads to functional difficulties. Negligence with regards to oral health care observed in patients suffering from schizophrenia or other psychotic disorders results from cognitive deficits which appear in the course of schizophrenia. Cognitive deficits make it difficult for these patients to care for their dentition status.

Antipsychotics and other medications used in the treatment of schizophrenia, such as antidepressants and mood stabilizers, may result in saliva secretion disorders or drug-induced mouth dryness (xerostomia), contributing to caries development [11]. Moreover, schizophrenic patients with a history of drug-related adverse effects, such as life-threatening neuroleptic malignant syndrome (NMS), or who have undergone electroconvulsive therapy (ECT) can be more suspicious not only of psychiatrists but also of other specialists, including dentists [12].

## Case presentation

A 30-year-old schizophrenic patient decided to visit the dentist office because of a toothache. The patient complained of burning sensation and dryness in her mouth and sleep disturbances. One week prior to the visit, the patient had stopped taking antipsychotic drugs prescribed by a psychiatrist, which she had taken at the following doses: aripiprazole 30 mg/day, perazine 100 mg/day, and carbamazepine 800 mg/day. She admitted to having taken the drugs irregularly. The patient either discontinued the treatment for several days or had taken double doses on subsequent days ‘to feel better faster’. The patient had poor insight into the illness, did not see a need for regular treatment of schizophrenia, and reported depressive mood caused by ‘chips in the teeth’. She had a history of neuroleptic malignant syndrome caused by risperidone treatment, therefore she was unwilling to take antipsychotic drugs, or she reduced the dosage by herself, being afraid of adverse events. During oral cavity and dental examination at the dentist office, the patient was diagnosed with circular and root caries, thick dental plaque, oral mucositis in the form of ulcerations, mucosal redness and dryness, as well as spontaneous mucosal lacerations. Food residues were also detected. Silness-Löe plaque index (PI) amounted to 2.29 and approximal plaque index (API) by Lange was 100% [13,14]. It was also found that her tongue tended to stick to the palate due to saliva hyposecretion. This condition caused difficulties in eating and bolus forming, affected her taste perception, and resulted in oversensitivity to the temperature of drunk liquids, as well as reduced appetite. In addition, the patient complained of halitosis (*fetor ex ore*), speaking difficulties, as well as throat dryness and soreness. The patient underwent sialometry which measured stimulated and unstimulated saliva secretion. Saliva hyposecretion was diagnosed, and it amounted to 0.5 ml/min following unstimulated saliva secretion and to 0.7 ml/min following stimulated saliva secretion (chewing of a gum). Unstimulated and stimulated saliva secretion was tested threefold within 6 min, 2 h after breakfast. With regards to numerous conditions concerning the patient’s dentition, she received periodontal treatment. The treatment involved supra- and subgingival plaque deposits removal (scaling) together with periodontal pocket irrigation and root debridement in order to reduce plaque deposits (bacterial biofilm) and to accelerate the cell healing process. An invasive treatment involved teeth 16, 17, and 33 with third degree root caries, tooth 26 with fourth degree caries, and due to esthetic reasons, tooth 43 with second degree root caries. Third degree dental caries were processed and filled with glass-ionomer cement (Ketac Molar®, 3 M ESPE, St. Paul, MN, USA). For a deep cavity of tooth 26, an additional self-hardening calcium hydroxide base (Life®, Kerr Corporation, Orange, CA, USA) was used. For esthetic reasons, caries on labial surfaces of teeth 33 and 43 were filled with complex material using the sandwich technique (Herculite®, Kerr Corporation, Orange, CA, USA). Following the treatment, the exposed root surfaces of all the teeth were covered with 35% CHX varnish (EC40™ Biodent®, Certichem, Nijmenen, Netherlands) for preventive reasons. Afterwards, the patient was once more instructed how to keep good oral cavity hygiene and how to perform routine oral hygiene activities correctly. It was recommended that the patient precisely remove food residues and dental plaque with the use of a nonabrasive fluoride toothpaste (Elmex, Colgate GABA Therwill, Switzerland) after each meal, and rinse her mouth with alcohol-free mouthwashes containing 0.1% chlorhexidine (Eludril Classic, Pierre Fabre Medicament, Warsaw, Poland). For xerostomia treatment, a pilocarpine-based formulation was applied with splitting daily dosage of 10 mg/day. During policarpine treatment, the patient was instructed to drink more liquids, in the amount of 2 l/day, to prevent from drug-induced dehydration. Following policarpine treatment and full recovery from the oral infection and dental caries, it was recommended that the patient applies artificial saliva based on mucin, carboxymethylcellulose, and glycerine. In addition to periodontal treatment, the patient underwent psychiatric treatment and participated in psychoeducation on schizophrenia. Psychiatric treatment involved administering olanzapine at a dose of 25 mg/day, which allowed for improvement in patient’s mental health. The psychotic symptoms (delusions and hallucinations) ceased, and cognitive functions returned to normal. The patient underwent individual cognitive behavioral therapy (CBT) and participated in psychoeducation. After 4 weeks, the patient had her oral hygiene status once more examined with the use of the indexes previously applied. Silness-Löe plaque index amounted to 0.58 and approximal plaque index to 23%, which indicated significant dental plaque reduction. After 12 weeks, all the teeth were covered again with 35% CHX varnish (EC40™ Biodent®, Certichem, Nijmenen, Netherlands). The amount of *Streptococcus* mutant was significantly decreased in saliva and dental plaque. Effectiveness of EC40 was confirmed by the direct contact test (DCT) (Nunclon Delta Surface; Nunc, Roskilde, Denmark). The DCT was used to assess the antimicrobial activity of the CHX varnishes [15].

With regular dental visits, continuous psychiatric care and participation in psychoeducation, the patient regained control over her health status and did not feel anxious about the therapy any more. The patient started compliance with the doctors and began to visit her dentist, psychiatrist, and psychologist on a regular basis.

## Discussion

Saliva is responsible for maintaining homeostasis within the oral cavity. It protects against mechanical and chemical injuries and has antibacterial, antifungal, digestive, and anticarious effects [16].

Saliva secretion disorder may cause excessive mouth dryness as a result of saliva hyposecretion or salivation as a result of saliva hypersecretion. Dry mouth, xerostomia (sialopenia, hypoptyalism, hyposalivation), can be caused by actual saliva hyposecretion (true xerostomia) or subjective dry mouth sensation (false xerostomia) in people with normal saliva production. Quantity and composition of secreted saliva are controlled by the sympathetic and parasympathetic nervous systems, nerve terminals which are located in salivary glands [17,18].

Drug-induced side effects are most frequently responsible for saliva secretion disorders [19,20]. In the described case, the patient reported saliva secretion disorders after taking antipsychotic drugs, such as aripiprazole, pernazine, and risperidone. Risperidone not only disturbed saliva secretion but also caused neuroleptic malignant syndrome, which is an idiopathic, life-threatening condition mainly related to the use of antipsychotic drugs. Less common, but also possible, is NMS, which may appear as a result of lithium monotherapy or the treatment with antidepressants (clomipramine, dezipramine), amantadine, carbamazepine, l-dopa, anticholinergic agents, ganciclovir, iron formulations, metoclopramide, as well as oral contraceptives. Neuroleptic malignant syndrome may develop even after a single dose of the drug which is administered as premedication before surgery, although within a therapeutic range [21]. According to the classification of mental disorders, Diagnostic and Statistical Manual of Mental Disorders, Fourth Edition, Text Revision (DSM-IV-TR), in order for NMS to be diagnosed, it is necessary to detect severe muscle rigidity and high fever directly after the last dose of antipsychotics has been taken. Also, at least two additional symptoms are required to occur, such as diaphoresis, dysphagia, shivering, incontinence, consciousness disturbance - from hypersomnia to coma, mutism, tachycardia, increased or labile blood pressure, leukocytosis, or increased creatine phosphokinase levels (CPK) [22]. Fever, progressing consciousness disturbance, changes in the autonomic nervous system activity, and extrapyramidal symptoms in patients treated with antipsychotics indicate that they are developing NMS. In the case of the described patient, a history of neuroleptic malignant syndrome was the reason for her discontinuing antipsychotic treatment or increasing the medication doses too rapidly.

Fluctuations in antipsychotic serum levels of the patient caused xerostomia. Inconsistent daily dosing of antipsychotics led to stimulation of sympathetic postsynaptic alpha-1 adrenergic receptors. As a result, water and electrolytes secretion increased. Beta-1 adrenergic receptors stimulation resulted in elevated secretion of enzymes and proteins with saliva. Activation of somatodendritic alpha-2 adrenergic receptors inhibited saliva secretion. The parasympathetic system is responsible for regulation of salivary secretion, which controls the process through M1 and M3 muscarinic receptors. Acetylocholine and vasoactive intestinal peptide released at axon terminals of parasympathetic neurones increase secretion of water and electrolytes after binding to muscarinic receptors of secretory cells in the salivary glands. Activation of the sympathetic nervous system inhibits the parasympathetic nervous system through alpha-2 adrenergic receptors in preganglionic parasympathetic neurons and at peripheral terminals of postganglionic parasympathetic neurones.

Dry mouth occurs during stimulation of the sympathetic system, e.g., when a patient feels anxiety. This reduces secretion of serous saliva and stimulates production of mucous (thick) saliva through activation of stimulating beta-receptors. Substances affecting the receptors of the autonomic nervous system disturb saliva secretion. Antagonists of alpha-1 adrenergic and muscarinic receptors, agonists of alpha-2 adrenergic receptor, and noradrenaline reuptake inhibitors cause dry mouth, while antagonists of alpha-2 receptor and agonists of muscarinic and alpha-1 adrenergic receptors increase saliva secretion [23].

## Conclusion

Schizophrenic patients often neglect not only psychiatric treatment but also their somatic health status. In antipsychotic treatment, it is important to detect drug-induced complications early, which may occur in the form of saliva secretion disorders, exacerbation of the disease symptoms, ineffectiveness of the applied pharmacotherapy, or life-threatening conditions, such as NMS. Schizophrenic patients who have experienced drug-induced adverse effects may be reluctant to compliance with their psychiatrist. In addition, side effects of antipsychotics, including dry mouth, may encourage mentally ill patients to visit their dentist. Therefore, a good cooperation of psychiatrists with other specialists in the scope of treatment for a particular patient not only allows for selection of the most comprehensive method of treatment yet also minimises drug-induced adverse effects.

### Consent

Written informed consent was obtained from the patient for publication of this case report. A copy of the written consent has been made available for review by the Editor-in-Chief of this Journal.

## Abbreviations

CPK: creatine phosphokinase
DSM-IV-TR: Diagnostic and Statistical Manual of Mental Disorders, Fourth Edition, Text Revision
NMS: neuroleptic malignant syndrome
